# Chronic Inflammatory Affections of the Palms and Soles

**Published:** 1909-04-17

**Authors:** 


### Dermatology.

### CHRONIC INFLAMMATORY AFFECTIONS OF THE PALMS AND SOLES.

Hyperkeratosis, or hypertrophy of the horny layers of the epidermis of the palms and soles is seen sometimes as a congenital malady, affecting generally several members of one family or of several generations of a family. Palmar and plantar hyperkeratosis is also a manifestation of chronic arsenical poisoning, such as may occur when arsenic is given in medicinal doses over long periods. It is part, too, of the rare eruption known as pityriasis rubra pilaris. In each of these conditions the hypertrophy of the horny layers is the main feature, and the whole of the palmar or plantar surface is involved.

In the affections which we are now about to consider, the thickening of the epidermis and the scaling are due, not to a simple hyperkeratosis, but to an active but imperfect cornification—parakeratosis—the result of an inflammatory condition in the deeper parts of the epidermis and the derma. The lesions are generally limited to a part only of the area of the palms or soles, and they may affect the palms only, or the soles only, or one palm or one sole. There are three common skin diseases which may produce eruptions of this nature on the palms and soles, namely, eczema, syphilis, and psoriasis. Each of these diseases, when affecting these regions, is liable to lose its characteristic features, owing to the different anatomical conditions of the skin of these parts, and to give rise to similar circumscribed areas whose main features are inflammatory redness, the formation of thick scales, and fissuring of the parts affected. For this reason there is often considerable difficulty in distinguishing them from one another, and one may frequently have to rely upon other evidence than that given by the actual lesion, such as, particularly, the presence of a more typical eruption of eczema, of syphilis, or of psoriasis on other parts of the body. If these are present the diagnosis is easy; if they are not, a very careful examination may be required. For the sake of treatment an accurate diagnosis, especially between syphilitic and non-syphilitic eruptions, is most important.

In eczema of the palms and soles the same pathological processes go on as in eczema elsewhere, but, owing to the thickness of the horny layers, the serous exudation cannot easily break through, and consequently it soddens the horny layers and separates them from the deeper epidermal layers in the form of largish scales. There is thus produced a circumscribed patch, which shows red where the inflamed œdematous tissues are partially denuded of horny layers, and which elsewhere is covered by thickened loosely adherent scales. Generally the red œdematous epidermis becomes deeply fissured down to the inflamed derma. Often, on careful examination of the skin beyond the main scaly patch, one can observe little collections of tiny deep-seated vesicles beneath the horny layers, and this appearance at once gives the clue to eczema. There is also intense itching, and the eruption is generally symmetrical, affecting both palms or both soles.

Syphilis of the palms and soles may occur as part of a general secondary papular or papulo-squamous eruption, where some of the flat papules have happened to appear on the palms or soles, and these produced scaly circumscribed patches. But the more important type, and that which needs to be distinguished from eczema, is a later manifestation, and what is known as a "recurrent secondary," or a tertiary lesion. As in eczema of the palms or soles, there is the same sharply circumscribed, red, scaly patch. But there are these differences: at the margin of the patch there is generally a more or less marked infiltration of the deeper tissues, sometimes actually nodular, sometimes only made out on careful examination; the whole condition is usually less acutely inflammatory, more indolent, and of a dull coppery hue rather than a bright red; there are no outlying deep-seated pin-head sized vesicles; the patches do not itch; they are often confined to one palm or to one sole. Another important fact is that these palmar and plantar syphilides are very frequently associated with syphilitic leucoplakia of the tongue, if the patient be a man who smokes. These syphilides are often very obstinate to treatment, and do not yield to the ordinary internal medication and local applications. Especially when associated with leucoplakia of the tongue, they are suitable cases for intra-muscular injections of grey-oil, under which treatment they do very well.

Psoriasis of the palms and soles is not uncommon in association with a general outbreak of psoriasis, and then its diagnosis is easy. Very rarely, however, it may occur without other parts being affected, and then it may be difficult to distinguish from eczema and from syphilis. The main difference is that in psoriasis the scales are more abundant and heaped up uniformly over the whole patch, not here and there detached to expose the inflamed epidermis as in the two latter eruptions. The scales, too, have the characteristic silvery aspect.

